# Salve for Hemorrhoids

**Published:** 1890-12

**Authors:** 


SALVE FOR HEMORRHOIDS.—The following is an excellent salve for hemorrhoids:

| | | |
|---|---|---|
| ℞. | Cocainæ muriat, - - - - | gr. xx. |
| | Morphinæ sulph., - - - - | gr. v. |
| | Atropinæ sulph., - - - - | gr. iv. |
| | Pulv. tannin, - - - - | gr. xx. |
| | Vaseline, - - - - - | ℥j. |
| | Ol. rosa, q. s. | |

M. Sig. Apply after each evacuation of bowels. Of course contents of bowels should be kept in soluble condition.

—*Archives of Pediatrics.*

